# Low Temperature Hydrophilic SiC Wafer Level Direct Bonding for Ultrahigh-Voltage Device Applications

**DOI:** 10.3390/mi12121575

**Published:** 2021-12-17

**Authors:** Wenting Zhang, Caorui Zhang, Junmin Wu, Fei Yang, Yunlai An, Fangjing Hu, Ji Fan

**Affiliations:** 1 State Key Laboratory of Advanced Power Transmission Technology, Global Energy Interconnection Research Institute Co., Ltd., Beijing 102209, China; zhangwenting200606@126.com (W.Z.); wujunmin2050@163.com (J.W.); yangsenji@163.com (F.Y.); yunlai_an@163.com (Y.A.); 2 MOE Key Laboratory of Fundamental Physical Quantities Measurement & Hubei Key Laboratory of Gravitation and Quantum Physics, PGMF and School of Physics, Huazhong University of Science and Technology, Wuhan 430074, China; zcr@hust.edu.cn

**Keywords:** SiC, plasma activation, low temperature, direct bonding

## Abstract

SiC direct bonding using O_2_ plasma activation is investigated in this work. SiC substrate and n^−^ SiC epitaxy growth layer are activated with an optimized duration of 60s and power of the oxygen ion beam source at 20 W. After O_2_ plasma activation, both the SiC substrate and *n*^−^ SiC epitaxy growth layer present a sufficient hydrophilic surface for bonding. The two 4-inch wafers are prebonded at room temperature followed by an annealing process in an atmospheric N_2_ ambient for 3 h at 300 °C. The scanning results obtained by C-mode scanning acoustic microscopy (C-SAM) shows a high bonding uniformity. The bonding strength of 1473 mJ/m^2^ is achieved. The bonding mechanisms are investigated through interface analysis by transmission electron microscopy (TEM) and energy dispersive X-ray spectroscopy (EDX). Oxygen is found between the two interfaces, which indicates Si–O and C–O are formed at the bonding interface. However, a C-rich area is also detected at the bonding interface, which reveals the formation of C-C bonds in the activated SiC surface layer. These results show the potential of low cost and efficient surface activation method for SiC direct bonding for ultrahigh-voltage devices applications.

## 1. Introduction

Power electronic devices have been widely applied in consumer electronics, electric vehicles, grid control and industrial applications [1]. Compared with silicon-based power devices, SiC is considered a promising material for next generation power devices for its wider band-gap, higher electric breakdown field, higher carrier saturation velocity, and higher thermal conductivity [2]. Therefore, SiC-based power devices display several advantages such as high compact factor, low power consumption, higher operation temperature and frequency, and simpler heat sink requirement [3,4]. Significant improvements in high quality SiC wafer fabrication have made SiC-based power devices, including Schottky diodes, bipolar junction transistors (BJTs), metal-oxide-semiconductor field effect transistors (MOSFETs), and junction field-effect transistors (JFETs), commercially available. In particular, the investigation of SiC insulated-gate bipolar transistors (IGBT) save been demonstrated by several researchers [5,6,7,8]. Benefiting from the high breakdown electric field (*E_bd_*), which is approximately 10 times higher than that of Si-based devices, the SiC-based devices can easily operate in an ultrahigh-voltage region larger than 10 kV [9,10,11]. Therefore, it is very suitable for large electric power system applications for which a large current operation condition is normally required. 

**Figure 1:**
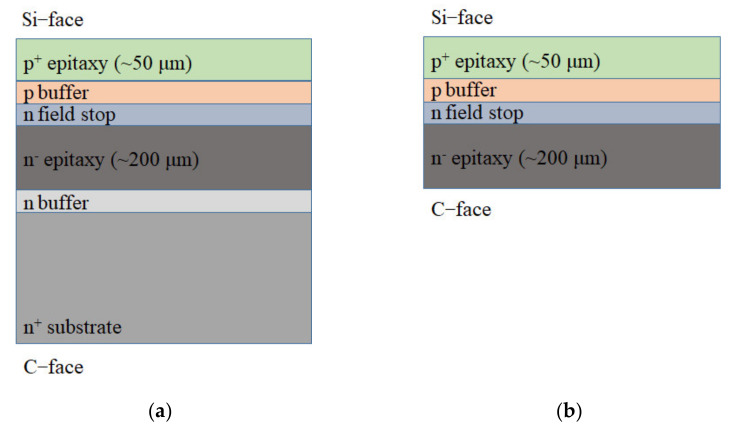
Process flow of the thick multi-layer SiC for the C-face *n*-channel IGBT: (**a**) growth of epitaxy layer on SiC substrate; (**b**) free-standing epi-wafer.

Figure 1 shows the typical process flow of a thick multi-layer SiC for the C-face *n*-channel IGBTs. First, the *n* buffer and thick *n*^−^ epitaxy layers were deposited on the Si-face of *n*^+^ substrate. Subsequently, *n*^−^ field stop, *p*^−^ buffer, and thick *p*^+^ layers were respectively grown on the Si-face. The thick *p*^+^ epitaxy layer is required to provide sufficient mechanical support for the ease of subsequent device fabrication processes (Figure 1a). In order to fabricate the structure on C-face epitaxy layer, the *n*^+^ substrate and *n*^−^ buffer layer were completely removed by polishing, resulting in a free-standing epi-wafer (Figure 1b) [10]. However, for a 3-inch wafer, the wafer bow after the final polishing can increase from <20 μm to ~70 μm, bringing additional difficulties for subsequent handling and processing. Moreover, for the purpose of high throughput and compatibility with commercial fabrication facilities, at least, the 4-inch wafer-level fabrication process is strongly required. If the 4-inch SiC-based device fabrication is applied, a free-standing epi-wafer with the thickness inferior to 300 μm will lead to a bigger handling and processing problem. If the Si-face of the epitaxy growth layer can be bonded with a SiC substrate before thinning and polishing, the problem of handling and processing caused by wafer bow can be effectively solved. However, the SiC–SiC bonding technique usually required an ultra-high annealing temperature and a long time [12,13]. Practical manufacturing requires the fabrication to be achieved at an adequately low temperature (typically 400 °C or below) for devices that are sensitive to high temperature processing owing to thermal budget limitation and the post-bonding thermo-mechanical stress control [14]. Pre-bonding surface activation has been proved as an effective method to ameliorate the bonding strength with a low annealing temperature [15]. SiC–SiC strong bonding at room temperature by modified surface activation bonding (SAB) with Fe–Si deposited layer or Si deposited layer have been demonstrated or proved to be feasible [2,3]. Although direct wafer bonding technique of SiC–SiC without any non-SiC interfacial have been reported [16,17], the SiC substrate bonding with the SiC epitaxy growth layer is still a major concern for the fabrication of ultrahigh-voltage devices.

In this paper, the prospects of a low temperature hydrophilic wafer level direct bonding between SiC substrate and *n*^−^ SiC epitaxy layer with O_2_ plasma activation are investigated. The surface activation is successfully achieved with an optimized duration and power of O_2_ plasma of 60 s and 20 W, respectively. The contact angle (CA) measurement with a goniometer and root mean square (RMS) surface roughness by atomic force microscopy (AFM) surface scanning. The 4-inch wafers are pre-bonded at room temperature, followed by an annealing process at 300 °C for 3 h. The bond strength measurement based on Maszara’s method is performed for the bonding quality assessment. Transmission electron micrograph (TEM) and energy dispersive X-ray spectroscopy (EDX) are employed to analyze the bonding mechanism. These results suggest the potential of low cost and efficient surface activation method for SiC substrate bonding with the SiC epitaxy layer for ultrahigh-voltage devices applications.

## 2. Experimental Details

**Figure 2:**
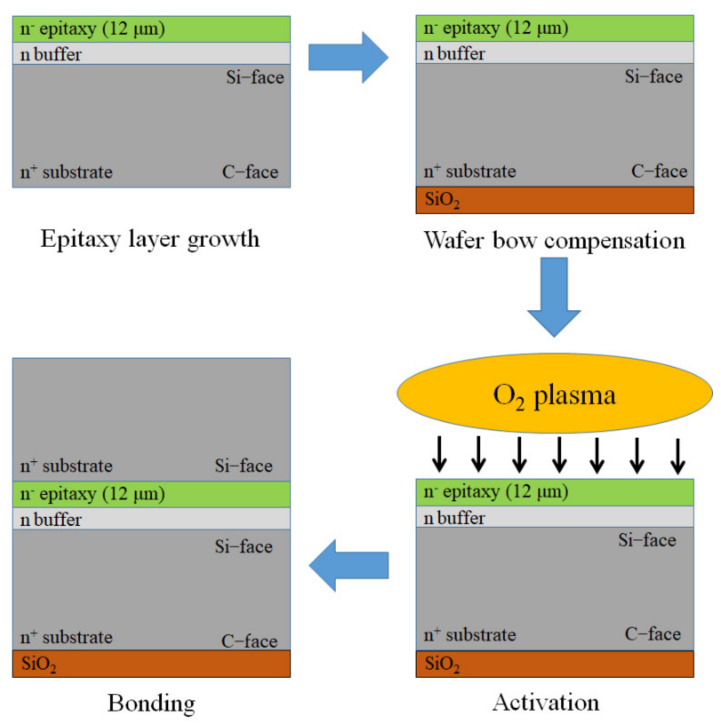
Schematics showing of bonding experiment.

The schematics of the bonding process is illustrated in Figure 2. Two SiC 4-inch wafers (*n*-type, 4° off-axis 4H-SiC wafer) with the thickness of 350 μm and 200 μm are utilized in the following bonding experiment. First, the *n* buffer layer and a 12 μm thick *n*^−^ epitaxy layer were grown on the 200 μm thick SiC wafer. In order to compensate the enormous wafer bow which was induced by the epitaxy layer growth, a SiO_2_ layer of 400 nm was deposited on the C-face of the SiC substrate. Then, the two wafers were cleaned in a conventional mixture of piranha solution (H_2_O_2_:H_2_SO_4_ = 1:1, by volume) and rinsed with DI water. To ameliorate the surface condition for direct bonding, the wafers were exposed to O_2_ plasma for 60 s with a power of 20 W. The O_2_ plasma energy is much higher than the energy of bonds such as C=C, C–O, O–H and Si–O. It is found that the bonding energies for C=C, C–O, O–H, Si–O are 620, 343, 465 and 368 kJ/mol, respectively [15,18], and the O_2_ plasma energy is 1175 kJ/mol [19]. Therefore, the hydrocarbon contaminations can be easily oxidized by O_2_ plasma, and CO, CO_2_ and H_2_O are formed as the byproducts. Meanwhile, the O_2_ plasma can also break down the covalent bond of Si-C at the bonding interface to form the hydrophilic interface. However, the time of exposure to O_2_ plasma must be carefully chosen, as prolonged exposure can increase the surface roughness that will lead to a bonding quality degradation. Subsequently, a DI water rinsing process was applied to hydroxylate the wafer surface with the hydroxyl (OH) group for hydrophilic bonding. The wafer with an *n*^−^ epitaxy layer and a blanket SiC wafer (Si-face) were brought into contact and bonded spontaneously using a commercial double-side aligner in room ambient. After pre-bonding, the wafer pairs were annealed at 300 °C in an atmospheric N_2_ ambient for 3 h.

## 3. Experiment Results and Discussions

### 3.1. Surface Activation

**Figure 3:**
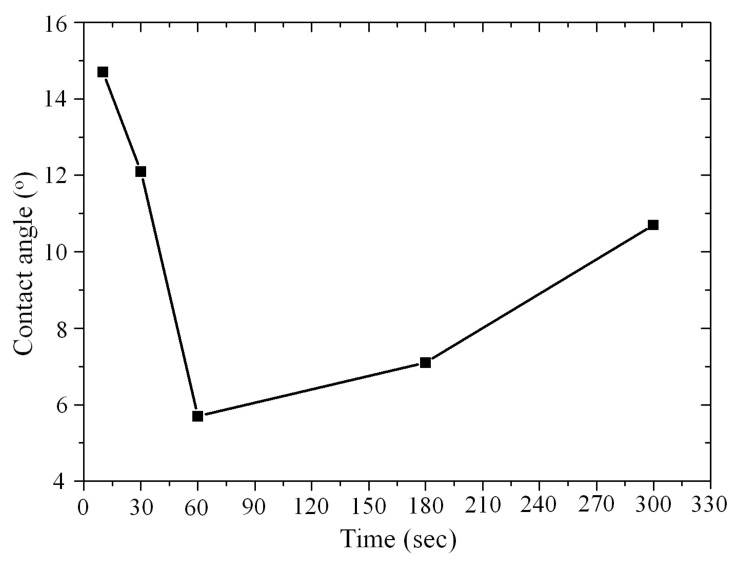
Contact angle versus plasma activation duration for a fixed plasma activation power of 30 W.

**Figure 4:**
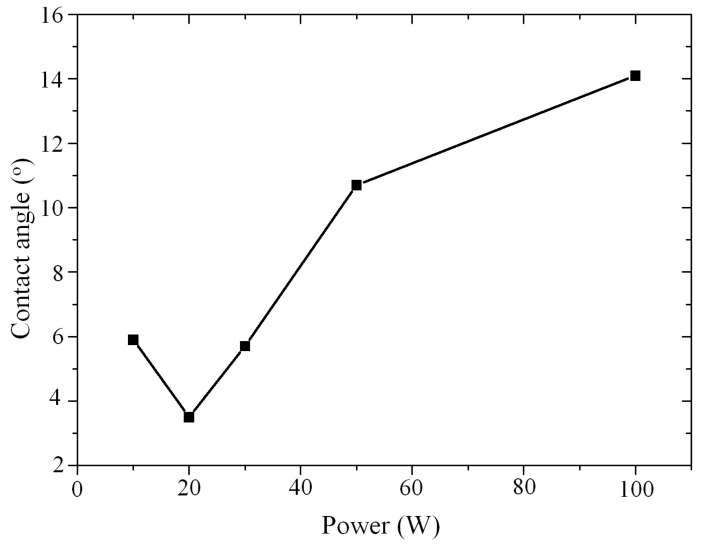
Contact angle versus plasma activation power for a fixed plasma activation time of 60 s.

In order to achieve a high bonding quality, direct bonding requires a critical hydrophilic surface between the two pairing wafers [20]. Thus, the activation conditions must be carefully optimized. First, the activation power was fixed at 30 W for the optimization of duration time. Figure 3 demonstrates the contact angle (CA) of the SiC samples versus duration time. It can be observed that the reverse effect on the contact angle due to prolonged exposure to energetic particles is an important consideration. When the exposure time is increased to 60 s and above, the wafer surface shows an increasing CA. Since the O_2_ plasma activation is a method using plasma bombardment, the wafer surface roughness will degrade after a long time exposure. The minimum contact angle (5.7°) was achieved when the exposure time was 60 s. According to this result, we fixed the plasma activation time at 60 s for the activation power optimization. Figure 4 demonstrates the contact angle as a function of the plasma activation power. The reverse effect was observed as well, with a minimum contact angle (3.3°) achieved when the activation power was 20 W. Therefore, the optimized plasma activation power and duration were 20 W and 60 s respectively.

**Figure 5:**
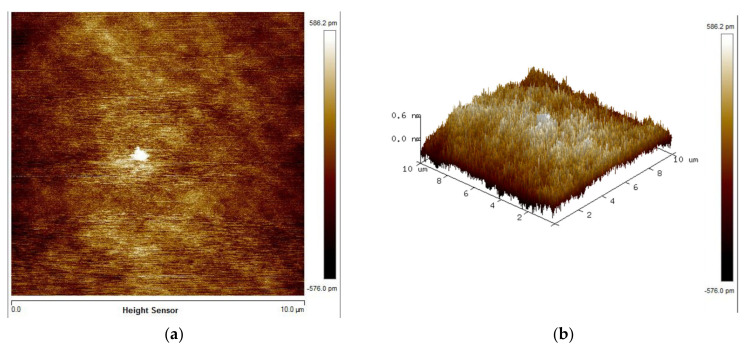
AFM images of SiC surfaces after O2 plasma activation: (**a**) 2D image; (**b**) 3D section image.

**Figure 6:**
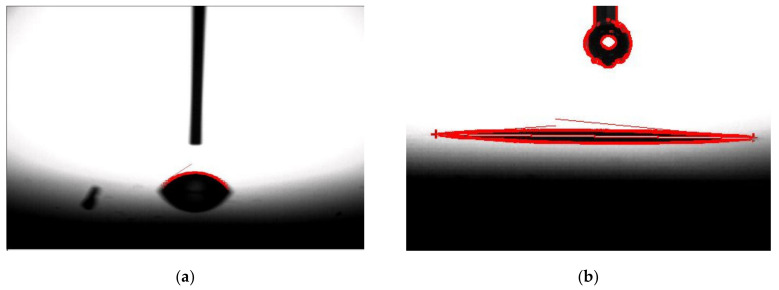
CA measurement: (**a**) Contact angle before activation; (**b**) Contact angle after activation.

Figure 5 shows the surface roughness for blanket SiC surface with activation using the tap mode of an atomic force microscope (AFM) and the image sensitivity is set to 1.26 µm/V. The RMS roughness is estimated based on the 10 μm × 10 μm AFM scan image. The RMS roughness of the control SiC wafer (without activation) is ~6.15 nm, and is improved to ~0.184 nm with the activation process by removing the surface hydrocarbon and other contaminants. It is below the roughness requirement of ~ 0.5 nm for a successful wafer-level direct bonding. The activation method was also applied to construct a hydrophilic bonding surface. As shown in Figure 6, the hydrophilicity of the exposed wafer was measured using water droplet contact angle measurement. Table 1 shows the contact angle and RMS roughness of the samples (to be bonded) before and after the activation. Table 2 shows the multiple measurements of the contact angle and RMS roughness of the SiC wafer after the activation. The control SiC wafer is less hydrophilic with a measured CA value of ~52.7°. When the CA decreases, more hydroxyl can be formed on the bonding surface. With the O_2_ plasma activation, the wafer shows a significant drop of CA to ~3.59°, resulting in a suitable hydrophilic surface. We have also optimized the activation duration and power for *n*^−^ SiC epitaxy growth layer, and the same process parameters are obtained. With the O_2_ plasma activation, *n*^−^ SiC epitaxy growth layer shows a decrease of CA to 7° and RMS roughness to 3.5 nm. Although these results are higher than those of SiC wafer, it is believed to be sufficient for hydrophilic bonding [21].

### 3.2. Bonding Uniformity and Bonding Strength

**Figure 7:**
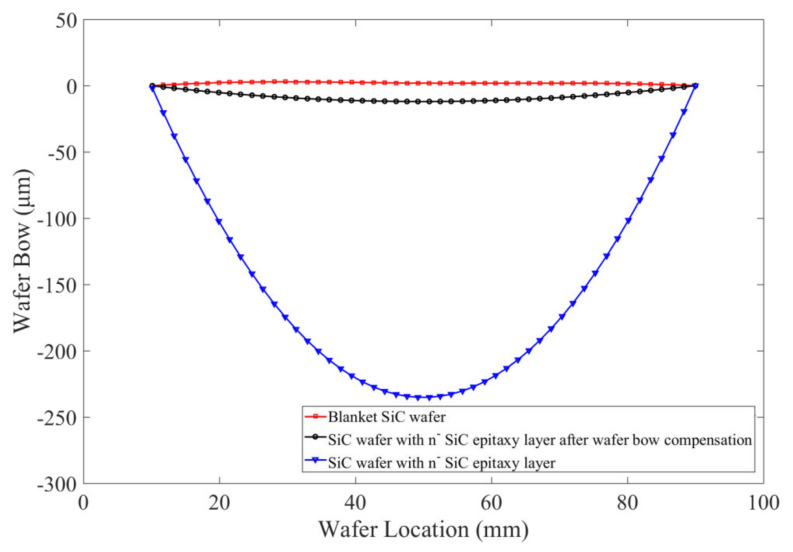
Pre−bonding wafer bow based on wafer curvature measurement.

**Figure 8:**
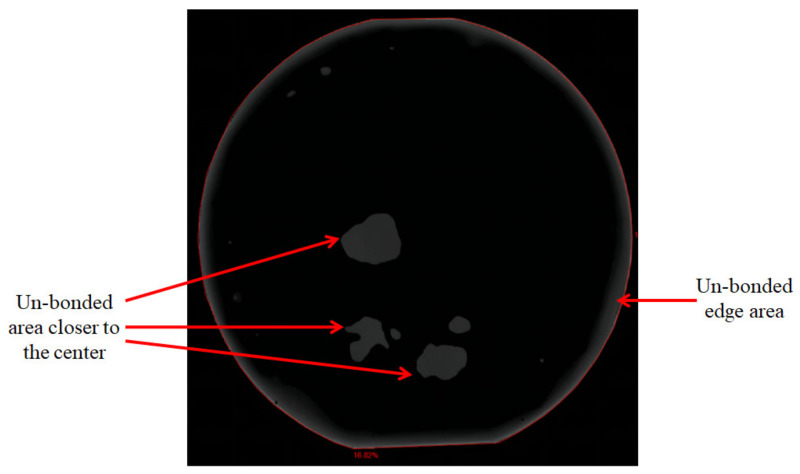
C-SAM scan image for bonded wafer pair.

The wafer bow detection based on wafer curvature measurement using a laser beam was performed before bonding. As shown in Figure 7, the SiC substrate exhibits a wafer bow of ∼3 μm, which is highly adaptive for subsequent direct bonding. However, the SiC wafer with *n*^−^ SiC epitaxy growth layer shows an increased wafer bow of ∼250 μm. This is a direct result of the huge difference in the coefficient of thermal expansion (CTE) between SiC substrate and *n*^−^ epitaxy layer. To the best of our knowledge, it is very difficult to achieve a high bonding uniformity with this enormous wafer bow. Therefore, the wafer bow compensation in the direct bonding technique is discussed in this study. Plasma Enhanced Chemical Vapor Deposition (PECVD) was used to deposit SiO_2_ on the back of *n*^−^ SiC epitaxy growth layer. The deposition forward power is 20 W. The gases used for deposition are N_2_O and SiH_4_. The deposition time is adjusted according to the film thickness calculated by the wafer bow of *n*^−^ SiC epitaxy growth layer. According to Figure 7, after a SiO_2_ deposition on the other side of the SiC wafer with *n*^−^ epitaxy layer, the wafer bow decrease to ∼12 μm, which was considered as a compatible deformation for direct bonding. C-mode scanning acoustic microscopy (C-SAM) was employed to examine the bonding interface of the wafer pairs. C-SAM examines reflected waves from interfaces external and internal to the sample, and can furnish a two-dimensional (area) description at a particular depth (Z). Figure 8 show the scan image of the bonded wafer pair, showing that the bonded pairs present a very high uniformity except for an un-bonded edge area and some small un-bonded areas across the wafer. The un-bonded edge area was most likely attributed to the trapped air due to a poor wafer handling, as we used tweezers for handling after activation. The small un-bonded areas closer to the center of the wafer were most likely caused by the wafer warpage. With the presence of these imperfections, the dicing yield of bonded wafers is about 85%.

After the SAM test, the Maszara’s crack opening method was employed to quantify the bonding strength by the surface energy of the bond. A razor blade with a thickness of 2*y* was inserted into the edge of the bonded wafer pair at the bonding interface. If the bonding interface at the edge is separated by the insertion of the razor blade, the crack will rapidly spread into the center of the wafer, and the crack length *L* can be measured from a customized IR imaging system. The surface energy of the bond can be estimated from the following relation [22]:

(1) $$\gamma =\frac{3Et^{3}y^{2}}{8L^{4}},$$

where *E* = 530 GPa, is the modulus of elasticity for single crystalline SiC, *t* is the thickness of the wafer, and *L* is the crack length. With a 300 °C annealing temperature, the bonded SiC substrate and *n*^−^ SiC epitaxy layer demonstrated a bonding strength of 1473 mJ/m^2^, which is comparable with the reported result of SiC–SiC substrate direct bonding [16]. Although this result is much less than the fracture energy between the Si and C-terminated ideal surfaces (considered as bulk SiC strength of 3400 J/m^2^ [23]), it is higher to sustain post-bonding processes such as mechanical grinding and polishing in micro-fabrication process (>1000 mJ/m^2^) [24].

### 3.3. Bonding Mechanism

**Figure 9:**
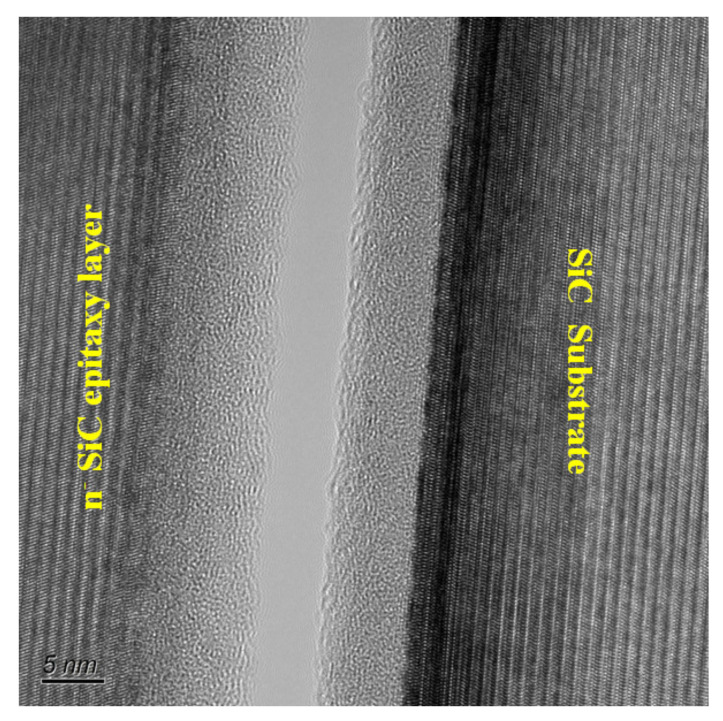
TEM image of the bonding interface of SiC wafer to *n*^−^ SiC epitaxy growth layer.

**Figure 10:**
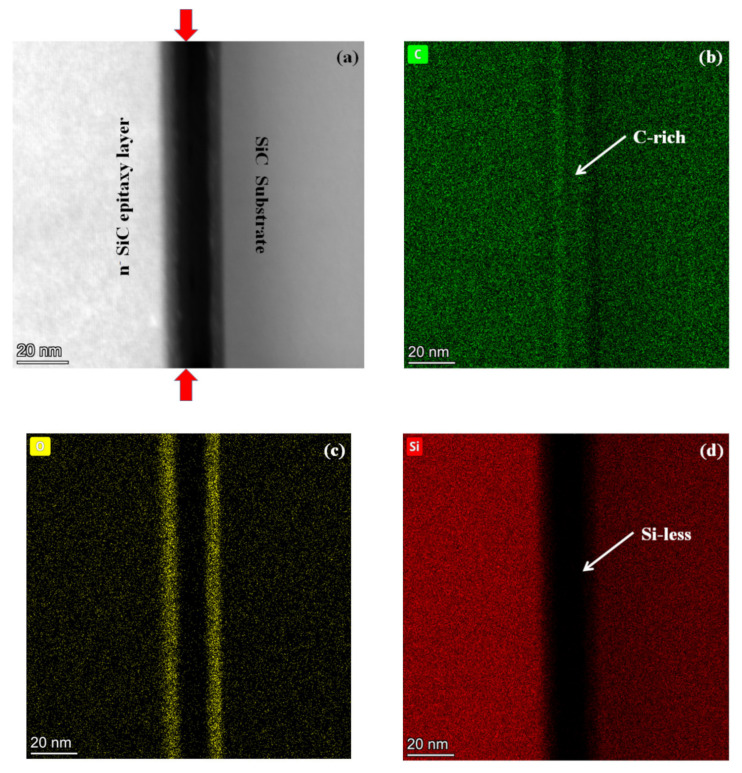
EDX element (C, O and Si) mapping of the bonding interfaces: (**a**) Mapping position at bonding interface; (**b**) C mapping; (**c**) O mapping; (**d**) Si mapping.

The bonding interface of the SiC wafer to *n*^−^ SiC epitaxy growth layer was further analyzed by TEM and energy dispersive EDX. Figure 9 shows the TEM image of bonding interface. According to the TEM image, it is believed that some reaction occurs at the bonding interface. It is noticed that there is a uniform amorphous layer (~5 nm) between the two interfaces. The formation of amorphous surface layer by surface activation may eliminate the effects of orientation. In this case, the hydroxyls can combine with the Si and C after activation, and thus a hydrophilic surface was obtained. The EDX images in Figure 10 show the element (C, O and Si) mapping of the SiC substrate to *n*^−^ SiC epitaxy growth layer bonding interface. It is observed that an amount of oxygen was found between the two interfaces. It is known that a large amount of hydroxyl result in a hydrophilic surface. In the Si direct bonding, the reaction of surface silanol (Si–OH) groups was enhanced and more hydrogen bonds were formed during annealing. At elevated temperature of 300 °C, the byproduct molecules of H_2_O gained more surface energy and diffused out of the bonding interface. As a result, strong siloxane (Si–O–Si) bonds were formed across the interface. It is believed that similar reaction occurs at the SiC substrate and *n*^−^ SiC epitaxy layer bonding interface. The covalent bonds, such as Si–O and C–O, were formed at the bonding interface, and the byproduct H_2_O diffuse out of the bonding interface. However, if there are only O- covalent bonds formed at the bonding interface, the bonding strength should reach to the bulk SiC strength. It can be found in Figure 10b that a C-rich area is shown in the bonding area. The oxygen content is less than that of Si at the bonding interface. According to earlier literature [3], the Si preferentially sputter during the plasma activation process, resulting in C-C residuum at the activated surface. This hypothesis was proved by the measured result in Figure 10d, which shows a Si-less area at the bonding interface. In this case, the Si–O and C–O bonds formed after annealing are insufficient to achieve a higher bonding strength than that of bulk SiC. The SiC substrate to *n*^−^ SiC epitaxy growth layer bonding strength (~1473 mJ/m^2^) presents in this study agrees with the hypothesis of SiC–SiC direct bonding. Since the epitaxy growth is less compact than the SiC substrate, Si atom is much easier to sputter during activation. Therefore, more activated C atoms could form a graphite-like structure at the surface, which leads to an aforementioned relative lower CA and higher RMS roughness [25,26,27].

## 4. Conclusions

O_2_ plasma activation was investigated for low temperature wafer-level hydrophilic SiC direct bonding. The O_2_ plasma activation was applied after a conventional piranha cleaning process. The CA and roughness results indicate that both the SiC and *n*^−^ SiC epitaxy growth layer surface were effectively cleaned and activated for subsequent hydrophilic direct bonding. Bonding uniformity measurement exhibits a high bonding quality with a short activation duration of 60 s and an activation power of 20 W. After the compensation of wafer bow, a high bonding uniformity of the direct wafer bonding of SiC substrate and *n*^−^ SiC epitaxy layer was achieved. The bonding energy measured with Maszara’s crack opening method was 1473 mJ/m^2^, which is sufficient for subsequent grinding or polishing process. The uniform amorphous layer at the interface shown in the TEM image indicates that the bonding can be achieved without orientation dependence. The EDX measurements shows a ~5 nm amorphous layer, indicating the bonding orientation independence between the SiC and *n*^−^ SiC epitaxy growth layer. The proposed bonding technique paves the way for wafer level SiC-based devices towards ultrahigh-voltage applications.

## Figures and Tables

**Table 1:** Contact angle and RMS roughness before and after activation.

	SiC Wafer		*n*^−^ SiC Epitaxy Growth Layer	
	Contact Angle	Rms Roughness	Contact Angle	Rms Roughness
Before activation	(52.7 ± 3.57)°	(6.15 ± 0.0986) nm	55.7°	9.5 nm
After activation	(3.59 ± 0.469)°	(0.184 ± 0.0351) nm	7°	3.5 nm

**Table 2:** Contact angle and RMS roughness of SiC wafer before and after activation.

	1	2	3	4	5
Contact angle before activation	51.8°	47.8°	53.5°	57.8°	52.4°
Contact angle after activation	3.3°	3.98°	3.67°	3.34°	3.67°
RMS roughness before activation	5.98 nm	6.20 nm	6.16 nm	6.22 nm	6.20 nm
RMS roughness after activation	0.131 nm	0.216 nm	0.201 nm	0.166 nm	0.206 nm
